# Supplementary material for: Assessing the stability of photon-counting CT: insights from a 2-year longitudinal study
Source: Eur Radiol. 2024 Dec 19;35(7):3721–8. doi: 10.1007/s00330-024-11244-y (PMC12165871; doi:10.1007/s00330-024-11244-y)
Supplement: Supplementary file 1 — Electronic Supplementary Material [file 330_2024_11244_MOESM1_ESM.pdf]

Assessing the stability of photon-counting CT: insights from a two-year longitudinal study

Electronic Supplementary Material (ESM)

Eur Radiol (2024) Liu LP, Pasyar P, Liu F et al.

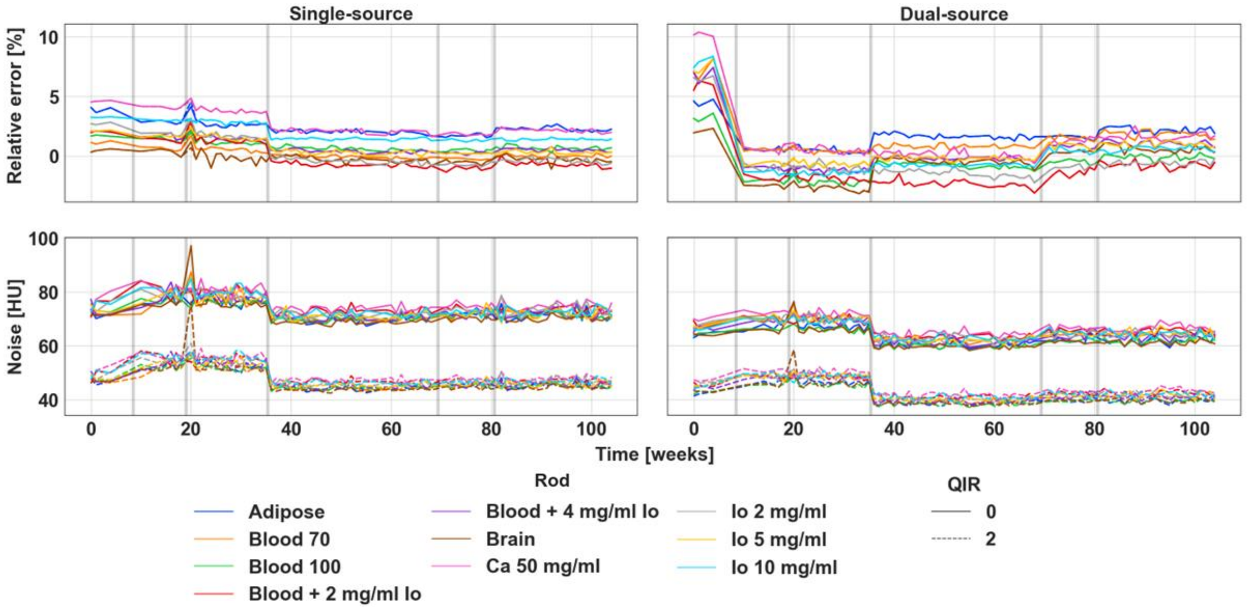

Figure S1. Stability of VMI 40 keV relative error and noise across time for single-source and dual-source modes. Gray bars in the figure indicate significant software (weeks 8, 35, 69) and hardware updates (weeks 8, 19, 80).

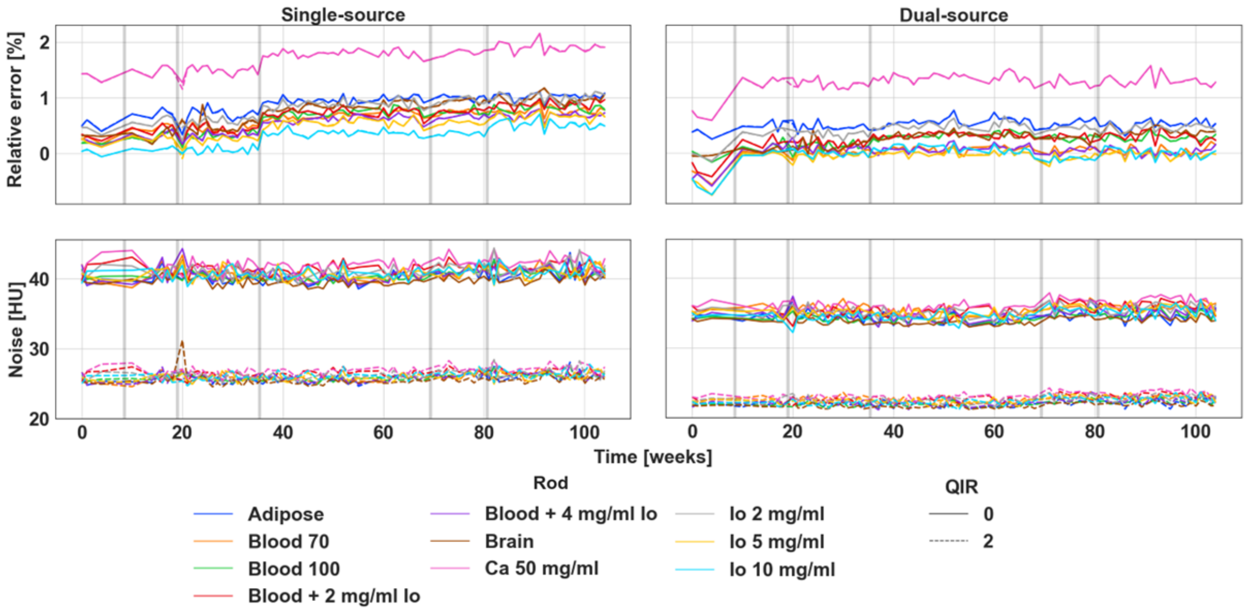

Figure S2. Stability of VMI 100 keV relative error and noise across time for single-source and dual-source modes. Gray bars in the figure indicate significant software (weeks 8, 35, 69) and hardware updates (weeks 8, 19, 80).

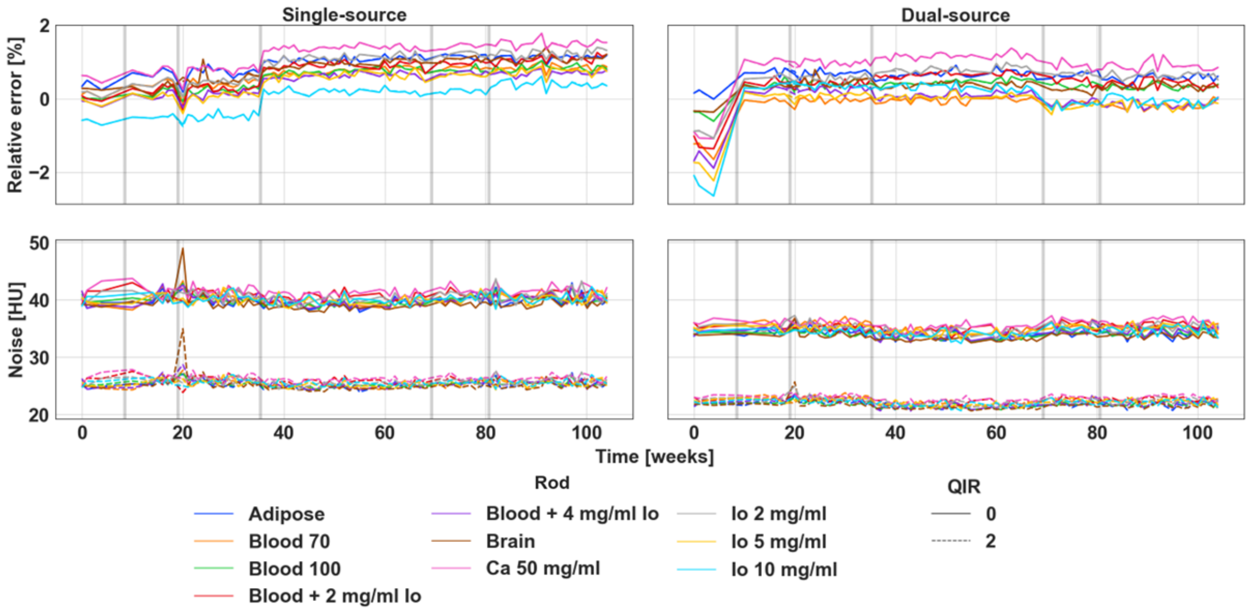

Figure S3. Stability of VMI 190 keV relative error and noise across time for single-source and dual-source modes. Gray bars in the figure indicate significant software (weeks 8, 35, 69) and hardware updates (weeks 8, 19, 80).
